# More Becomes Less: Management Strategy Has Definitely Changed over the Past Decade of Splenic Injury—A Nationwide Population-Based Study

**DOI:** 10.1155/2015/124969

**Published:** 2015-01-05

**Authors:** Kwan-Ming Soo, Tsung-Ying Lin, Chao-Wen Chen, Yen-Ko Lin, Liang-Chi Kuo, Jaw-Yuan Wang, Wei-Che Lee, Hsing-Lin Lin

**Affiliations:** ^1^Division of Trauma, Department of Surgery, Kaohsiung Medical University Hospital, Kaohsiung Medical University, Kaohsiung 807, Taiwan; ^2^Department of Emergency Medicine, Kaohsiung Medical University Hospital, Kaohsiung Medical University, Kaohsiung 807, Taiwan; ^3^Faculty of Medicine, College of Medicine, Kaohsiung Medical University, Kaohsiung 807, Taiwan; ^4^Graduate Institute of Medicine, Kaohsiung Medical University, Kaohsiung 807, Taiwan; ^5^Nutrition Support Team, Kaohsiung Medical University Hospital, Kaohsiung 807, Taiwan; ^6^Division of Gastrointestinal and General Surgery, Department of Surgery, Kaohsiung Medical University Hospital, Kaohsiung Medical University, Kaohsiung 807, Taiwan; ^7^Department of Surgery, Faculty of Medicine, Kaohsiung Medical University, Kaohsiung 807, Taiwan; ^8^Graduate Institute of Clinical Medicine, Kaohsiung Medical University, Kaohsiung 807, Taiwan

## Abstract

*Background*. Blunt spleen injury is generally taken as major trauma which is potentially lethal. However, the management strategy has progressively changed to noninvasive treatment over the decade. This study aimed to (1) find out the incidence and trend of strategy change; (2) investigate the effect of change on the mortality rate over the study period; and (3) evaluate the risk factors of mortality. *Materials and Methods*. We utilized nationwide population-based data to explore the incidence of BSI during a 12-year study period. The demographic characteristics, including gender, age, surgical intervention, blood transfusion, availability of CT scans, and numbers of coexisting injuries, were collected for analysis. Mortality, hospital length of stay, and cost were as outcome variables. *Results*. 578 splenic injuries were recorded with an estimated incidence of 48 per million per year. The average 12-year overall mortality rate during hospital stay was 5.28% (29/549). There is a trend of decreasing operative management in patients (*X* ^2^, *P* = 0.004). The risk factors for mortality in BSI from a multivariate logistic regression analysis were amount of transfusion (OR 1.033, *P* < 0.001, CI 1.017–1.049), with or without CT obtained (OR 0.347, *P* = 0.026, CI 0.158–0.889), and numbers of coexisting injuries (OR 1.346, *P* = 0.043, CI 1.010–1.842). *Conclusion*. Although uncommon of BSI, management strategy is obviously changed to nonoperative treatment without increasing mortality and blood transfusion under the increase of CT utilization. Patients with more coexisting injuries and more blood transfusion had higher mortality.

## 1. Introduction

Blunt spleen injury is a critical problem when a patient sustains trauma and is demonstrated to be the second most common solid organ injured with blunt abdominal trauma (BAT) [1, 2]. According to the National Trauma Data Bank Spleen Injury Mortality Rates of USA, blunt spleen injury leads to 7.0% to 22.7 % mortality depending on the grade of injury. However, previous epidemiological studies reported that the incidence of splenic injury varies with geographic locations and ethnic groups, with an overall mortality rate estimated at 6-7% [3–8].

Two decades ago, spleen injuries would usually be treated surgically if internal bleeding was suspected [9, 10]. Due to the improvement of speed, resolution, and interpretation experience in the CT scans, blunt spleen injury can be diagnosed and assessed without exploratory laparotomy [11]. In the study by Patrick et al., they reported that nonoperative management of injured children reduces the risks of receiving blood transfusion and decreases the length of hospital stay compared with aggressive operative intervention [12]. Recently, nonoperative management has been applied as the primary management strategy for these patients [6, 13–16]. However, the benefits of this application are not well documented. In addition, while overall mortality with blunt spleen injury was thought to gradually decrease under advanced intensive care and more nonoperative management [6, 17–20], this strategy has also become the mainstream treatment in our society [21–23]. We hypothesized that the change of the management strategy is based on improvement of diagnosis tools and the mortality and morbidity could be controlled within an acceptable range.

Therefore, using nationwide population-based data from Taiwan, the purpose of this study was to (1) find out the incidence and causes of strategy change; (2) investigate the effect of change on the mortality rate over the study period; and (3) identify risk factors for mortality in blunt spleen injury.

## 2. Patients and Methods

### 2.1. Data Source

The National Health Insurance (NHI) program in Taiwan was established in March 1995, covering more than 25 million enrollees, which represents approximately 98% of the population of Taiwan, by building a single-pipeline public insurance system for the entire population of Taiwan. The epidemiological analysis of blunt spleen injury was feasible due to the availability of hospitalization healthcare database, because almost all patients with blunt spleen injury in Taiwan are hospitalized to receive treatment. In the reports by the Taiwan National Health Research Institute, the Longitudinal Health Insurance Database contains the entire original claim data of 1,000,000 beneficiaries from 1997 to 2008, randomly sampled from the 25.68 million of those enrolled in the NHI program. No statistically significant differences were observed in age, gender, or average salary between the databank and the cohort of the Taiwan's population. This program provides a highly reliable database for researchers and various extracted datasets are available to researchers, and hundreds of published papers have used the NHI research databank as the basis for their studies. The databank consists of secondary data released to the public for research purposes, so the present study was exempted from full review by the Internal Review Board.

The criteria for inclusion were all patients with the International Classification of Disease-Clinical Modification, ninth revision (ICD-9-CM), codes 865.00 to 865.04 (spleen injury), and those who had received surgical intervention with the treatment code (70001B to 70006B) were identified. In NIH databank, there is one major code and four comorbidity ICD-9-CM codes (800.0 to 897.7 and 900.0 to 959.9) of final impressions for each patient. The coexisting injuries were indicated by those codes and then added together as the variation of coexisting injury. Patients who received blood transfusions were identified by code 11002C. Additional information such as gender, age, overall mortality, medical expenditure, major trauma, or relation to traffic accident was recorded. The exclusion criteria included (1) repeated admission without the diagnostic codes within 30 days, to prevent repeating the calculation; (2) patients who had any type of splenectomy unrelated to trauma; and (3) patients who had missing data.

**Figure 1:**
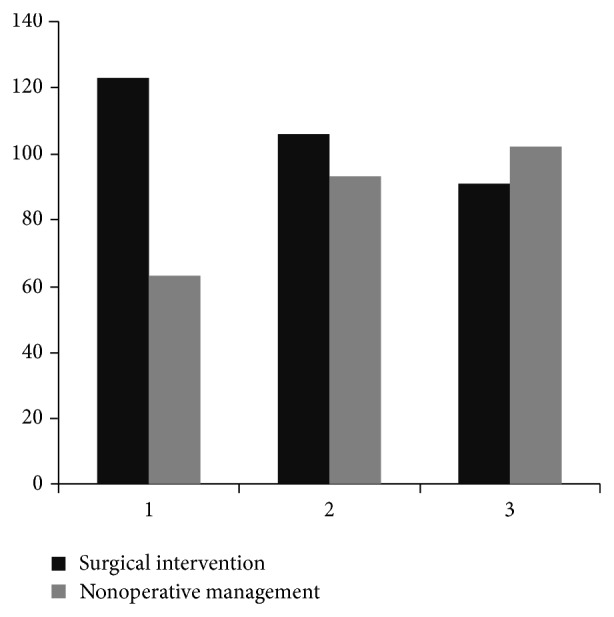
Incidence of surgical intervention in blunt spleen injury patients. There is a trend towards nonoperative management. 1 (1997–2000): *n* = 63/186; 2 (2001–2004): *n* = 93/199; 3 (2005–2008): *n* = 102/193.

To investigate sequential changes in the study period, annual data were summarized into three subperiods for the complete study population (Table 2) and for the subgroup of patients with mortality and medical expenditure (Figure 1).

### 2.2. Definitions of Major Trauma and Mortality

The total mortality rates during hospital stay were calculated as the number of deaths during hospitalization divided by the total number of spleen injuries annually. Patients who had an Injury Severity Score (ISS) equal to and more than 16 were categorized as major trauma [24–27] by the NHI after issuing a certification by administrated hospitals, with restricted recheck by peer reviewers by the Bureau of National Health Insurance, Department of Health of Taiwan, for insurance exemption.

### 2.3. Statistical Analysis

Basic characteristics were expressed as percentages. The chi-square test was used for categorical variables when compared between groups. For continuous variables, the Mann-Whitney *U* test (*U* test) was applied for comparison of two groups (mortality versus nonmortality patients) and the Kruskal-Wallis test for comparison of more than two groups (four subperiods of years). Data were presented as mean ± SD for continuous variables and as percentages for incidence rates. A trend analysis of conservative treatment was performed in patients with and without CT scans. All factors that were significant at *P* < 0.2 on bivariate analysis were entered in a stepwise logistic regression model to identify independent risk factors for death in this population and to estimate the adjusted OR and 95% CI. Where appropriate, mortality and other incidence rates are presented with 95% confidence intervals. A *P* value < 0.05 was considered significant. Statistical analysis was performed using standard statistical software (SPSS Version 17.0, SPSS, Chicago, IL).

## 3. Results

### 3.1. Basic Characteristics of the Study Population

During the study period, 578 patients with average age of 36.66 (±19.40) with blunt spleen injury were identified, with an incidence rate of 48 per million per year. Of these patients, the majority (73.18%, 423/578) were males. 19.03% (110/578) of the patients had major trauma and 32.53% (188/578) were caused by traffic accident. The overall incidence did not have differences during the 12-year study period (*χ* ^2^, *P* = 0.263). The average 12-year overall mortality rate during the hospital stay was 5.28% (29/549) (Table 1).

There were no differences in mortality between gender (*χ* ^2^, *P* = 0.928) and age (*U* test, *P* = 0.309). The overall mortality also had no difference between the presence or absence of traffic accident (*χ* ^2^, *P* = 0.860) or major trauma (*χ* ^2^, *P* = 0.815). Patients who expired had received significantly higher amounts of transfusion units in the hospitals than surviving patients (*U* test, 27.17 ± 29.14 versus 7.42 ± 13.79, *P* = 0.001); furthermore, they underwent surgery more frequently (*χ* ^2^, 79.31% [23/29] versus 54.09% [297/549], *P* = 0.008). The overall ICU stay had no significant impact on the mortality and survival rates (*U* test, 4.41 ± 14.22 versus 5.62 ± 10.29, *P* = 0.652); however, shorter hospital stay was noted with the mortality group (*U* test, 4.45 ± 9.73 versus 11.56 ± 9.34, *P* < 0.001). Patients who underwent surgery received more blood transfusions (10.76 ± 16.52 versus 3.48 ± 8.02, *P* < 0.001) (Table 1).

### 3.2. Stratification for Study Years

**Figure 2:**
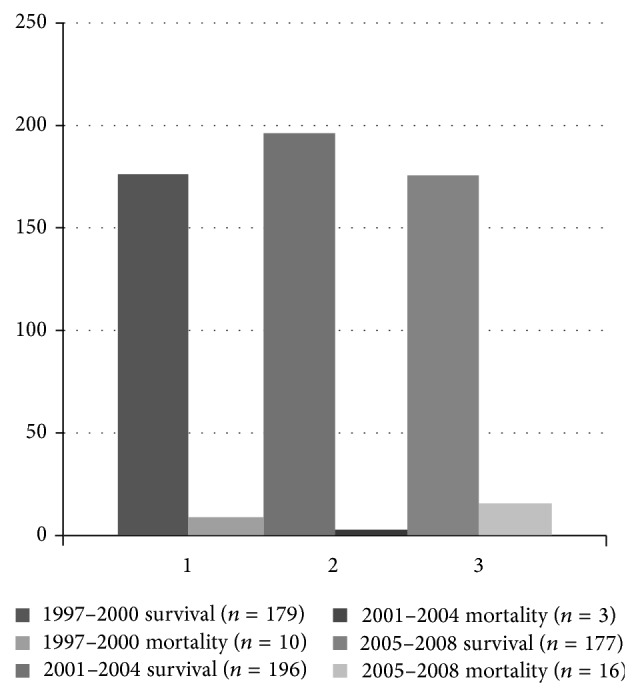
Incidence of mortality in blunt spleen injury patients.

The basic characteristics for the overall blunt spleen injury patients stratified into three subperiods according to the study year are displayed in Table 2. There was an increasing trend of blunt spleen injury caused by traffic accident and major trauma associated with more injuries. The utilization of CT scans was also on the rise. However, operation rate had decreased during the study period (Figure 1). Medical expenditure also demonstrated an increasing trend (Table 2). The mortality rate varied between the subperiods (Figure 2).

### 3.3. Predictors of Mortality from Multivariate Analysis

Eight variables were entered into the logistic regression model with results displayed in Table 3. The following variables were included as independent risk factors for mortality with blunt spleen injury: CT scans obtained, subperiod, amount of blood transfusion, and coexisting injuries. The following variables were excluded by the model: age, gender, major trauma (ISS > 16) [24–27], and operation versus nonoperative management. For this model, the percentage of accurately predicted cases was 95.0%. The Hosmer-Lemeshow statistic indicated a good fit without reaching significant value (*P* = 0.183).

## 4. Discussion

Blunt spleen injury is considered a lethal injury among various entities after BAT. According to European study, the incidence of blunt spleen injury is low, but it accounts for significant mortality [8]. In the previous studies of the United States, the overall mortality of blunt spleen injury was shown to vary from an average of 8.2% to 13% from 1981 to 2000 [28]. There are fewer large studies on the incidence and mortality of blunt spleen injury in Asia. In our study, the incidence of blunt spleen injury was not common (8.33 per million per year) and the mortality was low, with injured patient numbers being consistent every year. During each year of our data, the mortality rate in Taiwan remained at approximately 5% only.

Splenic injury was considered a common and major injury in abdominal contusion. We found that spleen injury is not common and lethal in Taiwan. We believed the EMT's transport time was less than 30 minutes to a capable hospital for definite management which may be a main reason for the lower possibility of mortality. Besides that, the trauma mechanisms are different compared to other countries [1, 8], in which our injuries are mostly caused by lower-velocity collisions [29]. In our study, blunt splenic injuries were almost included without exception by a nationwide population-based data and the mortality would be lower compared to the study using trauma registration, in which those less severe patients unnecessary to be transferred to trauma centers might not be included.

The increasing utilization of CT scans is found in many countries (e.g., from 59.1% in 1987 to 89.4% in 2001) [9–11, 30]. In our study, there was also an increasing trend of using CT scans for the diagnosis, with the rate on the rise from 40.3% in 1997 to 52.3% in 2008. What is interesting is that we found the increasing use of CT scans was correlated with a decrease in the numbers of surgical interventions. The rate of surgical intervention decreased from 66.2% to 47.2% within the same time period. It revealed that the strategy of nonoperative management had been generally accepted after the popularity of CT scan. We believe that the reason for lower rates of operation [21–23, 28, 31] is due to the increasing use of the CT scans [32, 33]. With advancement in CT scans, clinicians would be able to evaluate the potential of nonoperative management or plan for alternative strategy including angioembolization, thus avoiding unnecessary laparotomy solely for uncertainty of internal bleeding. Therefore, the increasing use of CT scans had changed the decision making of the management with less surgical intervention.

In a previous study, patients with severe splenic injuries were shown to be at risk for exsanguinations, shock-induced multiple organ failure, or other hemorrhage-related complications [34]. Under such circumstances, patients would usually receive massive transfusion during resuscitation. In the study by Robinson III et al., blood transfusion is found to be a strong independent predictor of mortality in patients with blunt spleen injury after controlling for indices of shock and injury severity. They also found that transfusion-associated mortality risk is the highest in the subset of their patients who were managed nonoperatively [35]. However, there were no reports studied about whether patients treated with nonoperative management wound receive more blood transfusion or not during hospitalization. In our study, we also found that patients with more amounts of blood transfusion had higher mortality rates; however, there has been a marked less operative intervention without more blood transfusion in this study decade.

Mortality in blunt spleen injury is usually caused by uncontrolled hemorrhage shock; however, the coexisting injuries are also an important risk factor of the mortality. In our study, there were no differences in mortality between age, gender, and major trauma. However, those with less coexisting injuries had better survival rates, which indicated that death in patients with blunt spleen injury may be also determined by coexisting injuries such as other intra-abdominal injuries, chest injuries, or traumatic brain injuries. A further study may be focused on the association of mortality with which body regions in these patients.

Several limitations of this study deserve comments. First, in common with other studies using administrative databases, the data did not provide detailed information of laboratory and clinical data (Glasgow Coma Scale, vital signs, blood pressure, and Injury Severity Score). Second, the selection of variables was limited because the source was a secondary databank. It is also possible that other unmeasured variables, such as patient trauma mechanisms (e.g., assault, fall, and motor vehicle accident), may contribute to the differences in mortality. The database is not generated for academic research, and coding errors might exist. However, coding of blunt spleen injury with the ICD-9-CM is very specific and most of the patients were treated by surgeons, so that the bias may be minimized. In addition, the NHI regularly and randomly samples a percentage of cases from hospitals to verify the validity of diagnosis and quality of care through peer chart reviews using touring professional teams. In this study, it was impossible to grade the spleen injury because half of the patients did not obtain CT.

## 5. Conclusion

In our study, more attention has been paid to those patients who have coexisting injuries and more blood transfusion with the aim of having less mortality. This study provides a proof of a more popular using of modern medical devices less the invasive procedures of the blunt spleen injury management, without increase of mortality, LOS, blood transfusion, and medical expedience.

## Figures and Tables

**Table 1:** Basic demographic characteristics and outcomes of blunt spleen injury in patients with and without mortality (1997–2008).

Demographic characteristic, outcome	Total, 578 (100%)	Mortality, 29 (5%)	Survival, 549 (95%)	*P*
Age (yrs; mean ± SD)	36.66 ± 19.40	36.36 ± 19.11	42.38 ± 23.87	0.052
Male (%)	73.2	72.4	73.2	0.928
Injury Severity Score >16 (%)	19.0	20.7	18.9	0.815
Blood transfusion (unit; mean ± SD)	8.41 ± 15.51	27.17 ± 29.14	7.42 ± 13.79	<0.001
Surgical intervention (%)	44.6	79.3	54.1	0.008
Intensive care unit length of stay (days; mean ± SD)	4.47 ± 14.04	5.62 ± 10.29	4.41 ± 14.22	0.309
In-hospital length of stay (days; mean ± SD)	11.20 ± 9.49	4.45 ± 9.73	11.56 ± 9.34	<0.001
Other coexisting injuries (n; mean ± SD)	3.06 ± 1.43	3.72 ± 1.28	3.03 ± 1.43	0.011
CT obtained (%)	46.5	31.0	47.4	0.086
Traffic accident (%)	32.5	31.0	32.6	0.860

**Table 2:** Basic characteristics and outcomes of the blunt spleen injury, stratified according to their respective study years (*n* = 578).

Characteristic, outcome	1997–2000	2001–2004	2005–2008	*P*
*n*	186	199	193	
Age (yrs; mean ± SD)	34.44 ± 19.23	37.91 ± 19.28	37.51 ± 19.59	0.143
Male (%)	76.8	71.9	71.0	0.396
Injury Severity Score >16 (%)	10.2	22.6	23.8	0.001
Blood transfusion (unit; mean ± SD)	6.59 ± 8.53	9.44 ± 18.50	9.11 ± 17.17	0.665
Surgical intervention (%)	66.1	53.3	47.2	0.001
Intensive care unit length of stay (days; mean ± SD)	2.31 ± 3.03	6.41 ± 21.82	4.55 ± 9.17	0.001
In-hospital length of stay (days; mean ± SD)	10.94 ± 8.51	11.74 ± 10.24	10.91 ± 9.60	0.668
Other coexisting injuries (*n*; mean ± SD)	2.95 ± 1.46	2.88 ± 1.45	3.36 ± 1.34	0.002
CT obtained (%)	40.3	46.7	52.3	0.064
Traffic accident (%)	25.8	28.1	43.5	<0.001
Medical fees	89007 ± 91498	105922 ± 129534	123314 ± 202402	0.320
Mortality (%)	5.4	1.5	8.3	0.009

**Table 3:** Independent risk factors for the mortality after blunt spleen injury, derived from a multivariate analysis using an entered logistic regression model with forward variable selection.

Variable entered	Regression coefficient β	Odds ratio (e^β^; 95% confidence interval)	*P*
CT obtained	−0.93	0.40 (0.16–0.96)	0.026
Blood transfusion	0.04	1.04 (1.02–1.06)	<0.001
Other coexisting injuries	0.32	1.38 (1.04–1.90)	0.043
Trauma years			
1997–2000			
2001–2004	−1.70	0.18 (0.04–0.88)	0.035
2005–2008	0.46	1.58 (0.64–3.93)	0.323
Constant	−6.52		
